# Prognostic value of lncRNA AFAP1‐AS1 in breast cancer: a meta‐analysis and validated study in Chinese population

**DOI:** 10.1002/cnr2.1923

**Published:** 2023-11-02

**Authors:** Zhenxing Yang, Tao Huang, Chong Sheng, Kaijuan Wang, Yilin Li, Yajing Feng, Dandan Huo, Fujiao Duan

**Affiliations:** ^1^ Department of Medical Research Office the Affiliated Cancer Hospital of Zhengzhou University & Henan Cancer Hospital Zhengzhou China; ^2^ College of Public Health Zhengzhou University Zhengzhou China; ^3^ Department of Hospital Infection Management the First Affiliated Hospital of Zhengzhou University Zhengzhou China

**Keywords:** breast cancer, lncRNA AFAP1‐AS1, prognosis, system evaluation

## Abstract

**Background:**

Long non encoding RNA (lncRNA) plays a crucial role in breast cancer. However, the prognostic role of AFAP1‐AS1 in breast cancer remains unclear.

**Aims:**

To investigate the relationship between the expression of long non‐coding RNA actin filament‐associated protein1 antisense RNA1 (AFAP1‐AS1) and prognosis of breast cancer.

**Methods and Results:**

Meta‐analysis was performed to explore the correlation between AFAP1‐AS1 and breast cancer. The AFAP1‐AS1expression in patients with breast cancer tissue and adjacent normal tissue from 153 patients was determined by qRT‐PCR. Bioinformatics and Cox proportional‐hazards risk model were used to explore the relationship between expression of AFAP1‐AS1 and prognosis. The combined analysis revealed a significant correlation between AFAP1‐AS1 expression and both overall survival (hazard ratios, HR = 2.33, 95%Cl: 1.94–2.81, *p* < 0.001) as well as disease‐free survival/progression‐free survival (HR = 2.94, 95%CI: 2.35–3.67, *p* < 0.001). The relation between expression of AFAP1‐AS1 and breast cancer was determined in 153 breast cancer and adjacent normal tissues. The findings revealed a significantly higher AFAP1‐AS1expression levels in breast cancer tissues compared to adjacent normal tissues (*p* < 0.001). Additionally, patients exhibiting heightened levels of AFAP1‐AS1 expression were correlated with an unfavorable prognosis (HR = 2.35, 95%CI: 1.47–3.74, *p* < 0.001), which aligns consistently with the findings of the pooled analysis. The subgroup analysis of clinical characteristics revealed a significant association between high expression of AFAP1‐AS1 and TNM stage (HR = 1.72, 95%CI: 1.11–2.65, *p* = 0.015).

**Conclusion:**

This study demonstrated that AFAP1‐AS1 acts as an oncogene and may serve as a novel prognostic marker for breast cancer, particularly in the Chinese population.

## 1 INTRODUCTION

The incidence of breast cancer is highest among women, making it a prevalent malignancy. Recent studies have shown that breast cancer accounts for 24.5% of newly diagnosed cases of cancer in 2020 and contributes to 15.5% of female cancer‐related fatalities.^1^ Breast cancer is highly heterogeneous, which affects its clinical process.^2^ Differences in patient expression profiles lead to heterogeneity, which makes the malignant behavior of tumor tissue different and affects the prognosis and treatment strategy.^3^ In addition to surgery and radiotherapy, the main methods of treatment in internal medicine are chemotherapy, endocrine, targeted and immunotherapy. With the advancement of medical treatment, the ability to treat and diagnose breast cancer has been substantially improved. However, some patients are progressing rapidly and their prognosis is poor.

With advancements in genomics, molecular biology, and other fundamental disciplines, extensive investigations have been conducted to elucidate the molecular mechanisms underlying cancer. More and more evidence indicates that long non‐coding RNA (lncRNA) is the main regulatory factors of a series of biological behaviors and diseases.^4^ The lncRNA plays a crucial role in breast cancer, which affects the molecular regulation, proliferation and metastasis, metabolism and autophagy of cancer.^5^

The rapid progress in high‐throughput sequencing and bioinformatics has led to the revelation of a wealth of information pertaining to lncRNA. Reiche et al. identified more than 9500 lncRNA transcripts that were expressed differently between adenocarcinoma and normal tissues.^6^ The AFAP1‐AS1 gene, located in the 4p16.1 region of chromosome 4, encodes an antisense RNA that associates with actin filaments. It is an antisense lncRNA transcribed from *AFAP1*antisense strand, with a total length of 6810 Bases, there are two transcripts, including enst00000608442.2 and enst00000674004.1. AFAP1 encodes motor fiber‐related protein, which belongs to AFAP1 family members, including AFAP1like‐1 and AFAP1 like‐2/xB‐130.^7^ Exon 2 of AFAP1‐AS1 exhibits an overlapping arrangement with exons 14, 15, and 16 responsible for encoding the protein AFAP1.^8^ AFAP1‐AS1, a prototypical lncRNA, exerts pivotal functions in the initiation and progression of diverse cancer types, exhibiting expression in both the cytoplasm and nucleus of malignant tumor cells.^8^ There is a growing body of evidence suggesting the upregulation of AFAP1‐AS1, an antisense lncRNA, in various cancer types, including breast cancer,^9^ cervical cancer,^10^ pancreatic cancer,^11^ lung cancer,^12^ esophageal squamous cell carcinoma.^13^

The AFAP1 protein has been identified as a substrate of virus cancer gene‐dependent tyrosine protein kinase, and its strong association with breast cancer has been well established.^14^ Zhang et al. demonstrated that AFAP1‐AS1 accelerates the oncogenic potential of triple‐negative breast cancer (TNBC) through modulation of the Wnt/b‐Catenin signaling pathway.^15^ Furthermore, AFAP1‐AS1 has been demonstrated to promote resistance to trastuzumab in breast cancer.^16^ The latest study has revealed that AFAP1‐AS1 plays a crucial role in promoting the survival of primary cells in TNBC by suppressing mitotic mutations and enhancing the growth, migration, and invasion capabilities. Mechanistically, AFAP1‐AS1 triggers the phosphorylation of PLK1 protein, a kinase associated with mitosis. The upregulation of AFAP1‐AS1 in TNBC primary cells leads to an elevation in the expression levels of downstream genes involved in the PLK1 pathway, including CDC25C, CDK1, BUB1, and TTK. Notably, AFAP1‐AS1 also contributes to increased lung metastasis.^17^ AFAP1‐AS1 holds promise as a potential prognostic biomarker and therapeutic target for TNBC.

Due to the limited number of studies on the correlation between AFAP1‐AS1 and breast cancer, there remains inconsistency regarding the specific targets of AFAP1‐AS1 and their indicative roles in breast cancer prognosis and occurrence. To investigate the AFAP1‐AS1 expression and prognosis in breast cancer, we conducted a meta‐analysis and bioinformatics analysis, followed by verification of our correlation results using a follow‐up population.

## 2 MATERIALS AND METHODS

The present study was conducted based on the Meta‐analysis of Observational Studies in Epidemiology (MOOSE).^18^ To establish the clinical significance of breast cancer‐associated AFAP1‐AS1, this study followed the PICO principles for study design, which include population, intervention, comparison, outcome and research design.

This study has obtained ethical approval from the committee of Zhengzhou University. All subjects involved in the present study were informed and signed the informed consent form in the preparation stage before starting the study.

### 2.1 Database selection and retrieval strategy

A two‐stage search strategy was implemented. The initial step was to systematically search the original articles by searching Web of science, PubMed, EMBASE, Cochrane database, CNKI (Chinese databases), and Wanfang (Chinese databases) until June 27, 2023. The retrieval approach encompasses three distinct sets of terms: “tumor/tumour” and “cancer” and “neoplasm”; “breast cancer”, “breast tumor” and “breast carcinoma”; “long noncoding RNA AFAP1‐AS1” and “lncRNA AFAP1‐AS1”; “prognosis”, “clinical outcome”, and “survival”. Literature retrieval was conducted by generating all possible combinations of at least one term from each group.

### 2.2 Inclusion and exclusion criteria

The inclusion criteria were as follows: (1) cohort studies investigating the correlation between AFAP1‐AS1 expression and breast cancer with respect to overall survival (OS) and/or disease‐free survival (DFS) or progression‐free survival (PFS), (2) based on high and low AFAP1‐AS1expression, breast cancer was divided into two groups, (3) the hazard ratios (HRs) and 95% confidence intervals (CIs) were either provided or could be inferred from the available data, (4) published in either Chinese or English language. The exclusion criteria were as follows: (1) correspondence, critical reviews, systematic reviews and meta‐analyses, expert perspectives, and case studies, (2) duplicate publications, (3) lack of data available for reckon the HRs and the 95%CIs.

In case of any data duplication or partial duplication with published articles, preference will be given to recently published or larger sample studies during the selection process.

### 2.3 Data extraction

Studies that meet inclusion criteria were independently estimated by two authors (KJW and FJD). If there were differences in the extracted data, the third author (YJF) would determine the final determination.

Eligible studies were extracted with following terms: first author name, publication year, size of sample, follow‐up duration, pathological subtype, clinicopathological characteristic as well as outcomes related to OS and DFS or PFS. For OS, the point of starting was the time of diagnosis, and the others were the treatment time or operation day. When HRs and/or 95%CIs were unavailable, the method of Parmar^19^ and Tierney^20^ was used for estimation.

### 2.4 Patient samples

A total of 153 breast cancer and adjacent normal tissues were collected from the Affiliated Cancer Hospital and the First Affiliated Hospital of Zhengzhou University between January 2014 and April 2016. All subjects included in this study were Chinese Han women who had recently been diagnosed with breast cancer and had not received any preoperative radiotherapy or chemotherapy treatment The clinical characteristics data and detailed pathological records were extracted, including age, history of the family, histological grade (I–III), lymph node metastasis, tumor node metastasis (TNM) stage (I–IV), estrogen receptor (ER), progesterone receptor (PR) and human epidermal growth factor receptor‐2 (HER2). The samples obtained from surgery were promptly flash‐frozen in liquid nitrogen within a time frame of 5 min and subsequently stored at a temperature of −80°C. The OS rate of the included patients was followed up for a median of 38 months, it was reckoned from surgical time to death time or last follow‐up date. DFS was computed from date of beginning of randomization and disease recurrence or (for any reason) death. PFS definition was the time from randomization to progress or death.

### 2.5 Quantitative real‐time polymerase chain reaction (qRT‐PCR)

Tissues were flash‐frozen in liquid nitrogen, and total RNA was extracted using the Trizol RNA extraction kit (Invitrogen, CA, USA). Subsequently, DNase I treatment (Thermo Scientific, Waltham, MA, USA) was performed to eliminate any potential genomic DNA contamination. The RNA concentration and purity were assessed using an ultraviolet (UV) spectrophotometer (NanoDropND‐1000, NanoDrop Technologies, USA). The RNA underwent reverse transcription utilizing the PrimeScript Reverse Transcription Kit (Qiagen, Valencia, CA). Evaluate the purity and concentration of RNA using a UV spectrophotometer (NanoDropND‐1000, NanoDrop Technologies, USA). Reverse transcription of RNA using PrimeScript reverse transcription kit (Qiagen, Valencia, CA).

The qRT‐PCR reaction was performed using ABI 7500 system and SYBR Green PCR master mix (Applied Biosystems, Foster City, CA). The primers of lncRNA–AFAP1‐AS1 5′‐AATGGTGGTAGGAGGGAGGA‐3′(sense) and 5′‐CACACAGGGGAATGAAGAGG‐3′(antisense); GAPDH primers 5′‐GCACCGTCAAGGCTGAGAAC‐3′(sense) and 5′‐ ATGGTGGTGAAGACGCCAGT‐3′ (antisense). The primer sequences were designed utilizing Primer Premier 5.0 software in accordance with the target gene sequence. To confirm their specificity, PCR product dissolution curve analysis was performed using the Applied Biosystems 7500 Sequence Detection System (Thermo Fisher Scientific, Inc.). The thermal cycling conditions comprised an initial denaturation step at 95°C for a duration of 30 s, followed by 40 cycles of amplification at 95°C for a duration of 5 s, and subsequent annealing/extension at 60°C for a duration of 30 s. The relative expression of RNA was analyzed using the 2^−ΔΔCt^ method^21^ by the ABI software. The median value was utilized as the threshold to classify high and low expression levels of AFAP1‐AS1 in the cancer samples collected from 153 patients with breast cancer.

Meanwhile, the expression level of AFAP1‐AS1 was validated through Gene Expression Profile Interaction Analysis (GEPIA), an integrated network interaction system utilized for analyzing RNA sequencing outcomes from 9736 cancer and 8587 normal samples in GTEx and TCGA databases.

### 2.6 Statistical analysis

The pooled analysis was conducted using Review Manager 5.3 (Oxford, UK). Q‐tests and the I‐squared (*I* ^2^) were applied to explore the inter‐study heterogeneity. Based on the results of heterogeneity analysis, either a fixed effect or random effects model will be employed. If there is no substantial significant heterogeneity (*P*_heterogeneity_ ≥ 0.10 or *I* ^2^ ≤ 50%), the fixed effect model will be employed to assess the combined effect size. On the contrary (*P*_heterogeneity_ < 0.1 and *I* ^2^ > 50%), the random effect model is employed. The stratified analyses were performed based on different prognoses, statistical analysis methods, pathological type and cut‐off values. Begg's and Egger's tests were conducted using STATA 13.1MP (StataCorp LP, USA) to assess publication bias.

Student's t‐test was utilized to compare two or multiple groups, while continuous data were presented as mean ± standard deviation (SD). The association between expression of AFAP1‐AS1 and clinical characteristics was assessed using the Person's chi‐square (*χ* ^*2*^) test.

Based on the classification of AFAP1‐AS1 expression, a significant correlation has been established between AFAP1‐AS1 expression and breast cancer prognosis. The Kaplan–Meier (KM) approach employed to evaluate the survival curve, while the log‐rank test was utilized for inter‐group comparison. The Cox proportional hazard regression models were carried out to reckon the HRs and its corresponding 95%CIs, and adjusted according to prognostic factors and clinicopathological characteristics.

The *p*‐values were calculated using a two‐sided test, and statistical significance was defined as *p* < 0.05.

## 3 RESULTS

### 3.1 Meta‐analysis

#### 3.1.1 Literature retrieval and characteristics of included studies

A total of 493 records were retrieved based on the literature retrieval strategy. Eight articles were evaluated and further screened and finally confirmed (Supplemental Figure 1). After a comprehensive screening and identification of processes, seven articles^8^, ^17^, ^22^, ^23^, ^24^, ^25^, ^26^ were evaluated in full text and finally confirmed. The study countries were determined corresponding to sources of the subjects. The studies included in the present meta‐analysis spanned from 2016 to 2023. OS in 1295 patients and DFS in 884 patients were analyzed from China. The histological subtypes were specified in four eligible studies TNBC. The expression levels of AFAP1‐AS1 in tissue were assessed using qRT‐PCR to investigate its association with OS and/or DFS. AFAP1‐AS1 expression cut‐off value was taken as median or normal (Table 1).

**TABLE 1:** Clinicopathological features of the included studies.

Author	Year	Country	Ethnicity	Number		Histology	TNM stage	Sample	Assay	Follow‐up (Months)	Cut‐off	Outcome
				OS	DFS/PFS							
Yang et al^a^	2023	China	Asian	153	DFS,153	Breast cancer	I–IV	Frozen tissue	qRT‐PCR	60	Median	HR/SC
Cen et al^17^	2023	China	Asian	368	DFS,368	TNBC	I–III	Frozen tissue	qRT‐PCR	78	Median	HR/SC
Wu et al^22^	2021	China	Asian	155		TNBC	I–IV	Frozen tissue	qRT‐PCR	36	Median	HR/SC
Bi et al^23^	2020	China	Asian	125	DFS,125	TNBC	NA	Frozen tissue	qRT‐PCR	144	Median	SC
Cai et al^24^	2020	China	Asian	20		Breast cancer	NA	Frozen tissue	qRT‐PCR	60	Normal	SC
Zhang et al^8^	2018	China	Asian	238	DFS,238	TNBC	I–IV	Frozen tissue	qRT‐PCR	126	Normal	SC
Ma et al^25^	2018	China	Asian	160		Breast cancer	I–IV	Frozen tissue	qRT‐PCR	36	Normal	HR/SC
Xie et al^26^	2016	China	Asian	76		Breast cancer	I–IV	Frozen tissue	qRT‐PCR	60	Normal	SC

Abbreviations: DFS, disease free survival; OS, overall survival; PFS, progressive free survival; qRT‐PCR, quantitative real‐time PCR; SC, survival curve; TNBC, triple‐negative breast cancer.

^a^ The present followed up study.

#### 3.1.2 Evidence synthesis

**FIGURE 1:**
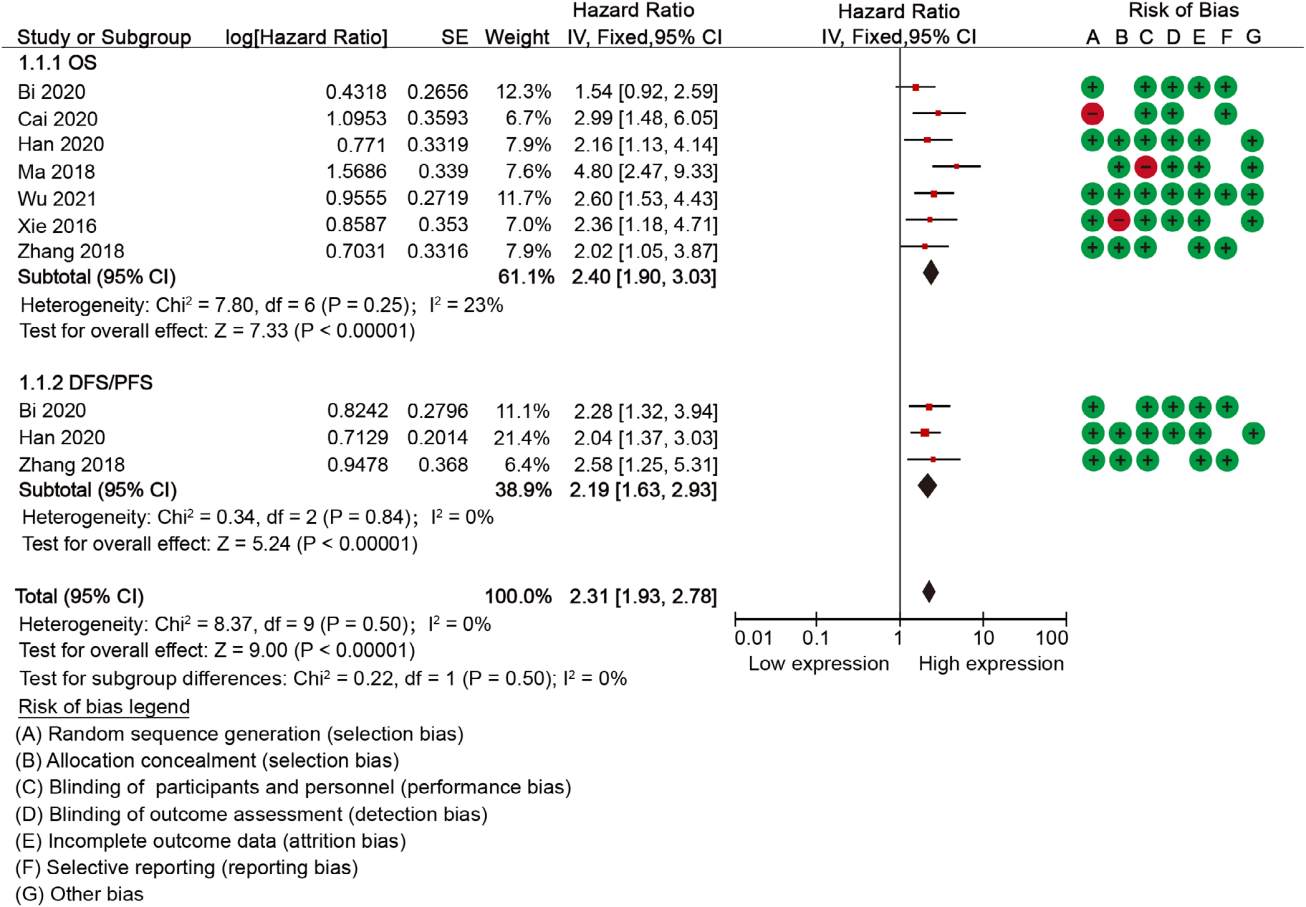
Forest plots depicting the association between AFAP1‐AS1 expression and prognosis.

According to Quality In Prognosis Studies (QUIPS) framework, the assessment of study quality for eligible studies was presented in Supplementary Table 1. The risk bias were presented in Figure 1. The scores of the Newcastle‐Ottawa Scale (NOS) reported in the study ranged from 5 to 9, with an average score of 7.25 as shown in Supplementary Table 2. Notably, a substantial proportion (87.5%, 7/8) of the included studies were classified as high‐quality.

The pooled OS analysis revealed a statistically insignificant inter‐study heterogeneity (*P*_heterogeneity_ = 0.35 and *I* ^2^ = 10%). Consequently, a fixed effect model was employed. The combined analysis demonstrated a significant correlation between AFAP1‐AS1 expression and OS (HR = 2.33, 95%Cl: 1.94–2.81, *p* < 0.001) (Table 2). For disease progression, the results showed the high levels of AFAP1‐AS1 expression predicted unfavorable DFS/PFS (HR = 2.94, 95%CI: 2.35–3.67, *p* < 0.001).

**TABLE 2:** Main results of pooled HRs in combined analysis.

Comparisons	Heterogeneity test			Summary HR (95%CI)	Hypothesis test		Model	Studies
	*Q*	*p*	*I* ^*2*^(%)		*Z*	*p*		
Total								
OS	8.02	0.33	13	2.33(1.94,2.81)	8.93	<0.001	Fixed	8
DFS/PFS	2.76	0.43	0	2.94(2.35,3.67)	9.53	<0.001	Fixed	4
OS								
Statistics analysis								
Log rank (KM)	1.02	0.80	0	2.36(1.88,2.97)	4.46	<0.001	Fixed	4
Multivariate analysis (Cox)	4.53	0.21	834	2.51(1.99,3.16)	7.80	<0.001	Fixed	4
Pathological type								
Breast cancer	3.90	0.42	0	2.71(2.06,3.57)	7.13	<0.001	Fixed	4
TNBC	1.99	0.57	0	2.07(1.63,2.62)	6.03	<0.001	Fixed	4
Cut‐off								
Median	2.91	0.41	0	2.16(1.70,2.74)	6.35	<0.001	Fixed	4
Normal	3.74	0.29	20	2.87(2.05,4.03)	6.11	<0.001	Fixed	4

Abbreviations: Cox, survival data from a multivariate Cox regression analysis; DFS, disease free survival; KM, survival data from a Kaplan–Meier curve; OS, overall survival; PFS, progressive free survival; SC, survival curve; TNBC, triple‐negative breast cancer.

Stratified analysis according to statistical approaches, pathological type and value of cut‐off showed that a significant association between expression of AFAP1‐AS1 and OS in patients with breast cancer (*p* < 0.001) (Table 2).

#### 3.1.3 Sensitivity analysis and assessment of publication bias

One‐at‐a‐time deletion method was employed for sensitivity analysis and recalculating the pooled HR. The stability of the results was indicated by the absence of significant changes in pooled HR (Data not shown).

Begg's (z = 1.61, *p* = 0.108) and Egger's test (*t* = 1.10, *p* = 0.314, 95%CI: −2.042 to 5.369) were applied to evaluate the bias of publication. Consequently, no indication of publication bias was detected, and the funnel plot exhibited a predominantly symmetrical shape (Data not shown).

### 3.2 AFAP1‐AS1 expression and prognosis

#### 3.2.1 AFAP1‐AS1 expression and clinicopathological characteristics

The clinical features encompass age, familial history, histological grade, TNM staging, lymph node status, ER/PR/HER2 expression, TNBC and triple‐positive breast cancer (TPBC), were summarized in Table 3. The results indicated no significant relationship between the expression level of AFAP1‐AS1 and family history, histological grade, ER, PR, HER2 status, TNBC versus TPBC in breast cancer (*p* > 0.05), but correlated with age (mean ± SD) (*p* = 0.005), TNM (*p* < 0.001) and lymph node metastasis (*p* = 0.005).

**TABLE 3:** The correlation between lncRNA AFAP1‐AS1 and clinical characteristics.

Characteristics	*N*	lncRNA AFAP1‐AS1 expression		*p* ^a^
		Low (*n* = 72)	High (*n* = 81)	
Age				0.681
Mean ± SD	153	48.17 ± 10.02	52.80 ± 9.89	0.005^b^
<50	56	29 (40.3%)	30 (37.0%)	
≥50	97	43 (59.7%)	51 (63.0%)	
Family history				0.200
No	137	67 (93.1%)	70 (86.4%)	
Yes	16	5 (6.9%)	11 (13.6%)	
Histological grade				0.067
I	44	16 (22.2%)	28 (34.6%)	
II	68	39 (54.2%)	29 (35.8%)	
III	41	17 (23.6%)	24 (29.6%)	
TNM stage				<0.001
I–II	85	55 (76.4%)	30 (37.1%)	
III–IV	68	17 (23.6%)	51 (62.9%)	
Lymph node metastasis				0.005
Negative	69	31 (43.1%)	53 (65.4%)	
Positive	84	41 (56.9%)	28 (34.6%)	
ER status				0.228
Negative	41	16 (22.2)	25 (30.9%)	
Positive	112	56 (77.8%)	56 (69.1%)	
PR status				0.741
Negative	38	17 (23.6%)	21 (25.9%)	
Positive	115	55 (76.4%)	60 (74.1%)	
HER2 status				0.744
Negative	49	24 (33.3%)	25 (30.9%)	
Positive	104	48 (66.7%)	56 (69.1%)	
Subtype				
TNBC	23	8 (53.3%)	15 (62.5%)	0.817
TPBC	16	7 (46.7%)	9 (37.5%)	

Abbreviations: ER, estrogen receptor; HER2, human epidermal growth factor receptor‐2; PR, progesterone receptor; TNBC, triple‐negative breast cancer; TPBC, triple‐positive breast cancer.

^a^ Person's chi‐square (*χ*)^*2*^test.

^b^ 2‐Sample t‐Test; TNM, tumor node metastasis.

#### 3.2.2 AFAP1‐AS1 expression in breast cancer tissue

**FIGURE 2:**
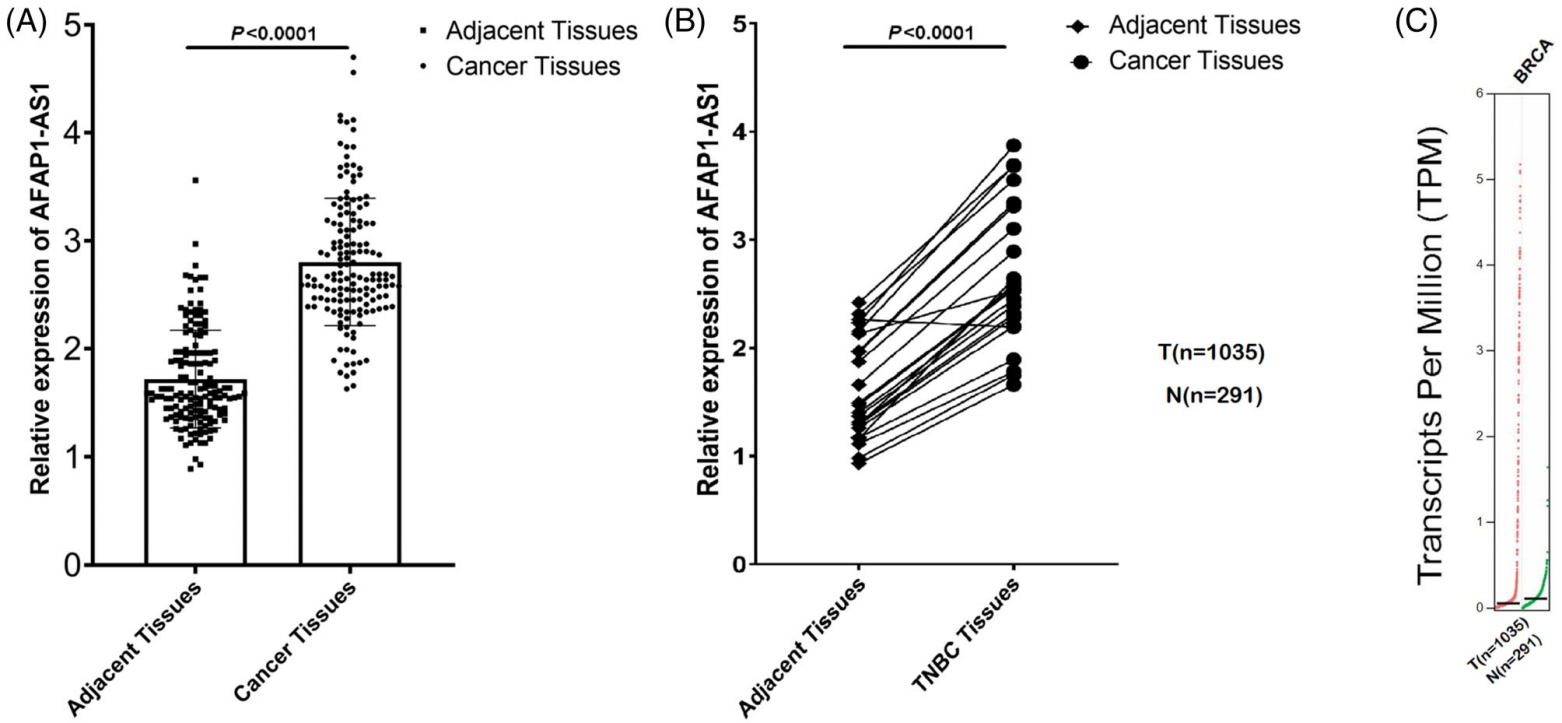
(A) Expression of AFAP1‐AS1 in breast cancer tissues and adjacent normal tissues. (B) Expression of AFAP1‐AS1 in triple‐negative breast cancer (TNBC) tissues and adjacent normal tissues. (C) Expression of AFAP1‐AS1 in breast cancer tissues (1085) and adjacent normal tissues (291) according to the log2 conversion of Transcripts Per Million (TPM). The red dots represent breast cancer tissue and the green dots represent adjacent normal tissue.

**FIGURE 3:**
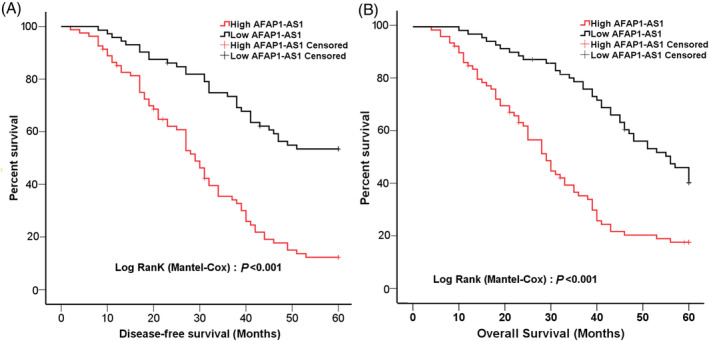
Expression of AFAP1‐AS1and prognosis in breast cancer. (A) Kaplan–Meier survival curves were generated to evaluate disease‐free survival in relation to AFAP1‐AS1 expression levels. (B) Kaplan–Meier survival curves were utilized to analyze the 5‐year overall survival rates based on AFAP1‐AS1expression levels.

The AFAP1‐AS1 expression was detected in 153 breast cancer and adjacent normal tissues using qRT‐PCR, indicating that the expressions of AFAP1‐AS1 in cancer tissue was increased markedly than that in adjacent normal tissue (Figure 2A,B). The bioinformatics tool “GEPIA” was applied to analyze 1085 patients with breast cancer and 291 normal tissues, indicating that the AFAP1‐AS1 expression in breast cancer tissue was significantly increased compared to adjacent normal tissue, as determined by Transcripts Per Million (TPM) (Figure 2C). The Kaplan–Meier survival analysis revealed a significant association between high AFAP1‐AS1 expression and poorer overall survival (OS) and disease‐free survival (DFS) in breast cancer patients, compared to those with low expression levels (*p* < 0.001, Figure 3A,B).

#### 3.2.3 Expression AFAP1‐AS1 and prognosis in breast cancer tissue

The significant factors identified in univariate analysis, including age, histological grade, TNM stage, lymph node metastasis, ER status and relative AFAP1‐AS1 expression levels were incorporated into a Cox proportional hazard regression model for further analysis. The results indicated that TNM (HR = 1.715, 95%CI: 1.110–2.649, *p* = 0.001) and expression of AFAP1‐AS1 (HR = 2.345, 95%CI: 1.470–3.742, *p* = 0.002) independently served as factors for OS (Table 4). The findings suggested that TNM (HR = 1.715, 95%CI: 1.110–2.649, *p* = 0.001) and AFAP1‐AS1 expression (HR = 2.345, 95%CI: 1.470–3.742, *p* = 0.002) were independent prognostic indicators for overall survival (Table 4).

**TABLE 4:** Univariate and multivariate Cox regression models were employed to analyze the overall survival of patients with breast cancer.

Factors	Categories	Univariate analysis		Multivariate analysis	
		HR (95%CI)	*p*	HR (95%CI)	*p*
Age	<50 versus ≥50	2.955(1.408,6.200)	0.004	1.063(0.703,1.608)	0.772
Family history	Positive versus Negative	1.639 (0.466,5.765)	0.441		
Histological grade	I–II versus III	2.357 (1.037,5.356)	0.041	1.092 (0.703,1.696)	0.695
TNM stage	I–II versus III–IV	3.699 (1.673,8.181)	0.001	1.715 (1.110,2.649)	0.015
Lymph node metastasis	Positive versus Negative	2.181 (1.046,4.544)	0.037	1.151 (0.756,1.752)	0.511
ER status	Positive versus Negative	0.381(0.172,0.842)	0.017	0.830 (0.540,1.275)	0.394
PR status	Positive versus Negative	1.130(0.478,2.673)	0.781		
HER2 status	Positive versus Negative	0.760 (0.343–1.685)	0.499		
AFAP1‐AS1 level	Low versus High	3.231 (1.503–6.943)	0.002	2.345 (1.470,3.742)	<0.001

Abbreviations: CI, confidence interval; ER, estrogen receptor; HR, Hazard ratio; HER2, human epidermal growth factor receptor‐2; PR, progesterone receptor.

## 4 DISCUSSION

With the aid of high‐throughput screening and second‐generation sequencing technology, a growing body of lncRNAs have been identified and annotated, thereby elucidating their biological functions in various diseases.^27^, ^28^ Although numerous studies have confirmed the involvement of lncRNAs in breast cancer pathogenesis, the repertoire of lncRNAs associated with this disease remains limited. The specific mechanisms of lncRNAs that affect breast cancer development have not yet been fully revealed, especially affecting the prognosis.

The high expression of AFAP1‐AS1 can affect the progression of various tumors, including nasopharyngeal carcinoma, renal cell carcinoma, liver cancer, colorectal cancer, etc.^29^, ^30^, ^31^ Silencing AFAP1‐AS1 in gallbladder cancer cells can upregulate E‐cadherin, downregulate Twist1 and Vimentin, and inhibit the migration and invasion of nasopharyngeal carcinoma cells by inhibiting the EMT process.^32^ In liver cancer cells, AFAP1‐AS1 can promote the progression of hepatocellular carcinoma by upregulating the positive Rho/Rac2 signaling pathway.^33^ Silencing AFAP1‐AS1 can inhibit the proliferation, migration, and invasion of tongue squamous cell carcinoma both in vivo and in vitro, and may become a potential diagnostic and prognostic biomarker and therapeutic target for tongue squamous cell carcinoma.^34^ However, the role of AFAP1‐AS1 in breast cancer remains to be verified.

The functions of AFAP1‐AS1 in different biological processes are mainly achieved by combining competitiveness with miRNA or interaction with protein through complex mechanisms. Numerous studies have demonstrated aberrant expression patterns of lncRNAs across various cancer types. AFAP1‐AS1 participates in multiple biological processes, including migration, invasion proliferation and apoptosis.^35^ The proliferation of breast cancer cells exhibited a significant correlation with both the clinical stage and tumor size.^36^ Therefore, further investigation into the relationship between aberrant expression of AFAP1‐AS1 and breast cancer progression will enhance our comprehension of the pathogenesis of breast cancer and facilitate identification of more reliable clinical therapeutic targets.

In the present study, relevant studies on breast cancer were retrieved from multiple online databases and a quantitative systematic review was conducted. The results demonstrated a significant association between high expression of AFAP1‐AS1 and unfavorable overall survival (HR = 2.33, 95%CI: 1.94–2.81, *p* < 0.001) as well as DFS (HR = 2.94, 95%CI: 2.35–3.67, *p* < 0.001). Meanwhile, we also detected the association between AFAP1‐AS1expression and breast cancer in subgroup analysis (statistics analysis, pathological type and cut‐off). After quantitative combination analysis, the association between the AFAP1‐AS1 expression and breast cancer was determined. The expression level of AFAP1‐AS1 was quantified in 153 breast cancer and adjacent normal tissues using qRT‐PCR. The findings revealed a significant elevation of AFAP1‐AS1 expression level in breast cancer tissues compared to adjacent normal tissues (*p* < 0.001). Patients exhibiting exhibited a significantly unfavorable prognosis (HR = 2.35, 95%CI: 1.47–3.74, *p* < 0.001), aligning consistently with the outcomes derived from our comprehensive meta‐analysis. Furthermore, we conducted an estimation of the potential correlation between expression of AFAP1‐AS1 and various clinical characteristics of patients. Our findings indicated that elevated levels of AFAP1‐AS1 were significantly related to TNM stage (HR = 1.72, 95%CI: 1.11–2.65, *p* < 0.015), while no significant correlations were observed with age, family history, histological grade, ER/PR/HER2 status, and TNBC versus TPBC. These findings suggested that AFAP1‐AS1 may serve as a valuable prognostic biomarker for breast cancer.

The underlying mechanism by which AFAP1‐AS1 modulates cellular biology remains elusive. The AFAP1‐AS1 transcript interacts with the AUF1 protein, thereby facilitating the translation of v‐erb‐b2 avian erythroblastosis leukemia viral oncogene homolog 2 (ERBB2) without exerting any influence on its mRNA abundance. The exon of AFAP1‐AS1 can induce trastuzumab resistance by binding with AUF1 and enhancing ERBB2 translation. Therefore, the expression level of AFAP1‐AS1 could potentially serve as a valuable prognostic indicator for predicting trastuzumab resistance and optimizing breast cancer treatment.^16^ The septin protein family comprises 14 members, among which Septin 2 is included.^37^ As a cytoskeleton protein, septins has numerous cellular functions including regulating cell migration and apoptosis.^38^, ^39^ AFAP1‐AS1 knocks down to inhibit the breast cancer cells progress through sponge‐based miR‐497‐5P and lower SEPT2.^24^

In mammalian cells, numerous studies have demonstrated the presence of both sense and antisense transcripts. Alterations in expression of antisense RNA can lead to changes in the expression of sense gene transcripts.^40^, ^41^ Yang et al. conducted a screening of total RNA from 7 HER2‐positive breast cancer tissues and adjacent normal tissues, resulting in the identification of 1382 differentially expressed lncRNAs, with AFAP1‐AS1 exhibiting the most significant expression.^21^ Ma et al. demonstrated a significant upregulation of AFAP1‐AS1 expression in breast cancer and cell lines, with elevated levels of AFAP1‐AS1 being associated with an unfavorable prognosis among breast cancer patients. Suppression of AFAP1‐AS1 expression significantly attenuates cell migration, invasion, and proliferation in breast cancer cell lines.^25^ Similar results were obtained by Dianatpour et al. Meanwhile, this study showed that AFAP1‐AS1 can act as a miR‐2110 sponge to regulate the progression of mouse breast cancer cells, and affect the occurrence of tumors. Therefore, the modulation of SP1 expression by AFAP1‐AS1 suggests a potential pivotal role of AFAP1‐AS1 in the therapeutic management of TNBC.^42^ Therefore, could serve as a promising biomarker for predicting the prognosis of breast cancer.^43^

In this study, certain limitations should be pointed out. Firstly, in the evidence‐based stage, all included study populations are from China, and studies from other genetic backgrounds may achieve different results, although the data showed no publication bias. Secondly, subjects are mostly from patients with early breast cancer, which has a certain effect on association between AFAP1‐AS1 expression and clinicopathological features. Thirdly, in house data have only a short follow up duration, which may have an adverse impact on the occurrence of prognostic outcomes. Finally, the molecular mechanism between AFAP1‐AS1 and prognosis is not involved and needs additional study.

## 5 CONCLUSION

In summary, a quantitative systematic review was conducted to investigate the correlation between AFAP1‐AS1 expression and breast cancer prognosis. The upregulation of AFAP1‐AS1 expression in breast cancer tissues has been confirmed. High expression of AFAP1‐AS1 in breast cancer is associated with a poor prognosis and advanced TNM stage of the disease. Our findings suggest that AFAP1‐AS1 acts as an oncogene and holds potential as a novel prognostic biomarker for breast cancer, particularly within the Chinese population. Revealing the function and underlying mechanism of AFAP1‐AS1 can offer valuable insights for clinicians to devise innovative strategies for breast cancer prevention and treatment in future studies.

## AUTHOR CONTRIBUTIONS

**Zhenxing Yang:** Conceptualization (equal); methodology (equal); software (equal); writing – review and editing (equal). **Tao Huang:** Project administration (equal); resources (equal). **Chong Sheng:** Data curation (equal); methodology (equal). **Kaijuan Wang:** Methodology (equal); validation (equal); writing – review and editing (equal). **Yilin Li:** Methodology (equal); writing – review and editing (equal). **Yajing Feng:** Data curation (equal); resources (equal). **Dandan Huo:** Formal analysis (equal); investigation (equal). **Fujiao Duan:** Conceptualization (equal); project administration (equal); supervision (equal).

## CONFLICT OF INTEREST STATEMENT

The authors have stated explicitly that there are no conflicts of interest in connection with this article.

## ETHICS APPROVAL AND CONSENT TO PARTICIPATE

This study was approved by the medical ethics committee of Zhengzhou University (ID: ZZUIRB 2017–0039) and adhered to the guidelines set forth in the Declaration of Helsinki.

## CONSENT FOR PUBLICATION

Prior to enrollment, all subjects provided written informed consent.

## Supporting information


**Supplementary Figure 1** The literature retrieval and study selection flowchart.
**Supplementary Table 1.** The eligible studies were evaluated for quality using the quality in prognosis studies (QUIPS) criteria.
**Supplementary Table 2.** The quality of cohort studies was assessed using the newcastle‐ottawa scale (NOS) for quality assessment of eligible studies.

## Data Availability

The data sets were used and/or analyzed during the present study are available from the first or corresponding author.
