# Supplementary material for: Prognostic value of lncRNA AFAP1‐AS1 in breast cancer: a meta‐analysis and validated study in Chinese population
Source: Cancer Rep (Hoboken). 2023 Nov 2;7(1):e1923. doi: 10.1002/cnr2.1923 (PMC10809272; doi:10.1002/cnr2.1923)
Supplement: Supplementary file 1 — Supplementary Figure 1 The literature retrieval and study selection flowchart. Supplementary Table 1. The eligible studies were evaluated for quality using the quality in prognosis studies (QUIPS) criteria. Supplementary Table 2. The quality of cohort studies was assessed using the newcastle‐ottawa scale (NOS) for quality assessment of eligible studies. [file CNR2-7-e1923-s001.docx]

**Supplementary materials**

Supplementary Figure 1 The literature retrieval and study selection flowchart.

Supplementary Table 1 The eligible studies were evaluated for quality using the Quality In Prognosis Studies (QUIPS) criteria.

Supplementary Table 2 The quality of cohort studies was evaluated using the Newcastle-Ottawa Scale (NOS) for quality assessment of eligible studies.


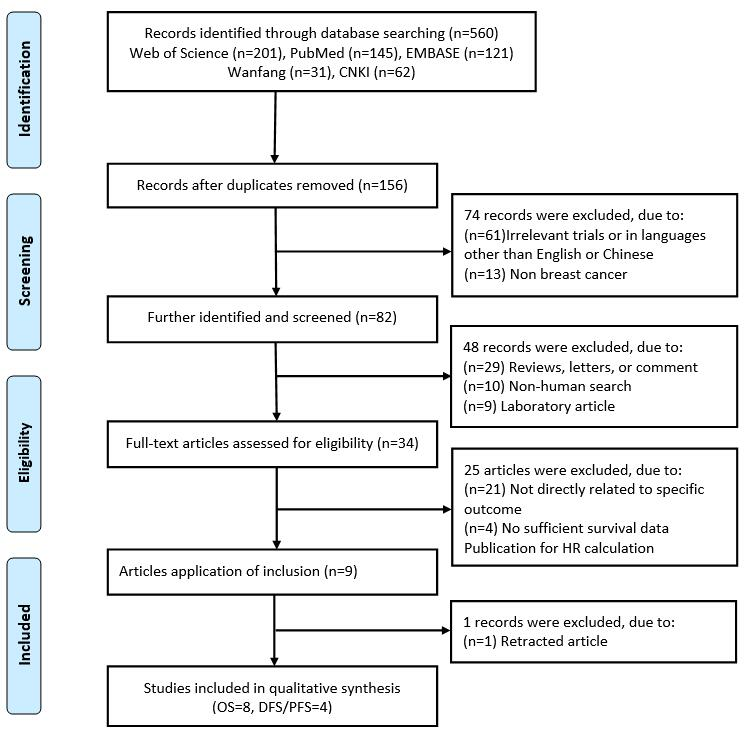


Supplementary Figure 1 The literature retrieval and study selection flowchart.

Supplementary Table 1 The eligible studies were evaluated for quality using the Quality In Prognosis Studies (QUIPS) criteria.

| **Study** | **Quality evaluation of prognosis study** | | | | | | **Total**  **Score^a^** | **Level of**  **Evidence^b^** |
| --- | --- | --- | --- | --- | --- | --- | --- | --- |
|  | **Study**  **Participation** | **Study**  **Attrition** | **Prognostic**  **Factor**  **Measurement** | **Outcome**  **Measurement** | **Study Confounding** | **Statistical Analysis and**  **Reporting** |  |  |
| Yang et al^a^ | Yes | Partly | Yes | Yes | Partly | Yes | **8** | **2b** |
| Cen et al[17] | Yes | Partly | Yes | Yes | Partly | Yes | **8** | **1b** |
| Wu et al[22] | Yes | Yes | Yes | Yes | Partly | Yes | **9** | **1b** |
| Bi et al[23] | Yes | Partly | Partly | Partly | Partly | Partly | **7** | **2b** |
| Cai et al[24] | Partly | Partly | Partly | Partly | Partly | Partly | **5** | **2b** |
| Zhang et al[8] | Yes | Partly | Partly | Partly | Partly | Partly | **7** | **2b** |
| Ma et al[25] | Yes | Partly | Yes | Yes | Partly | Yes | **7** | **2b** |
| Xie et al[26] | Partly | Partly | Yes | Partly | Partly | Partly | **7** | **2b** |

^a^ Quality assessment of included studies based on the Newcastle–Ottawa Scale (NOS).

^b^ The levels of evidence were estimated for all included studies with the Oxford Centre for Evidence Based Medicine criteria.

Supplementary Table 2 The quality of cohort studies was assessed using the Newcastle-Ottawa Scale (NOS) for quality assessment of eligible studies.

| **Study** | **Selection** |  |  |  |  | **Comparability** |  | **Exposure** |  |  |  |
| --- | --- | --- | --- | --- | --- | --- | --- | --- | --- | --- | --- |
|  | (score) |  |  |  |  | (score) |  | (score) |  |  |  |
|  | Representativenes  of the exposed cohort | Selection of the  non-exposed cohort | Ascertainment  of exposure | Outcome of interest was not present at  start of study |  | Based on the  design or analysis ^a^ |  | Assessment  of outcome | Follow-up long  enough for  outcomes to occur | Adequacy of  follow-up  of cohorts | Total  Score^b^ |
| Yang et al^a^ | 1 | 0 | 1 | 1 |  | 2 |  | 1 | 1 | 1 | **8** |
| Cen et al[17] | 1 | 0 | 1 | 1 |  | 2 |  | 1 | 1 | 1 | **8** |
| Wu et al[22] | 1 | 1 | 1 | 1 |  | 2 |  | 1 | 1 | 1 | **9** |
| Bi et al[23] | 1 | 0 | 1 | 1 |  | 2 |  | 1 | 1 | 0 | **7** |
| Cai et al[24] | 1 | 0 | 1 | 1 |  | 0 |  | 1 | 1 | 0 | **5** |
| Zhang et al[8] | 1 | 0 | 1 | 1 |  | 2 |  | 1 | 1 | 0 | **7** |
| Ma et al[25] | 1 | 0 | 1 | 1 |  | 2 |  | 1 | 1 | 1 | **7** |
| Xie et al[26] | 1 | 0 | 1 | 1 |  | 2 |  | 1 | 1 | 0 | **7** |

^a^When there was no statistical significance in the response rate between case and control groups by using a chi-squared test (*P* > 0.05), one point was awarded.

^b^Total score was calculated by adding up the points awarded in each item.
